# LIONS PREY: A New Logistic Scoring System for the Prediction of Malignant Pulmonary Nodules

**DOI:** 10.3390/cancers16040729

**Published:** 2024-02-09

**Authors:** Fabian Doerr, Annika Giese, Katja Höpker, Hruy Menghesha, Georg Schlachtenberger, Konstantinos Grapatsas, Natalie Baldes, Christian J. Baldus, Lars Hagmeyer, Hazem Fallouh, Daniel Pinto dos Santos, Edward M. Bender, Alexander Quaas, Matthias Heldwein, Thorsten Wahlers, Hubertus Hautzel, Kaid Darwiche, Christian Taube, Martin Schuler, Khosro Hekmat, Servet Bölükbas

**Affiliations:** 1 Department of Thoracic Surgery, West German Cancer Center, University Medical Center Essen-Ruhrlandklinik, University Duisburg-Essen, 45239 Essen, Germany; 2 Department of Anesthesiology and Intensive Care Medicine, Vinzenz Pallotti Hospital Bergisch Gladbach-Bensberg, GFO-Clinics Rhein-Berg, 51429 Bergisch Gladbach, Germany; 3 Clinic III for Internal Medicine, Faculty of Medicine, University Hospital Cologne, University of Cologne, 50923 Cologne, Germany; 4 Department of Thoracic Surgery, Helios Clinic Bonn/Rhein-Sieg, 53123 Bonn, Germany; 5 Division of Thoracic Surgery, Department of General, Thoracic and Vascular Surgery, Bonn University Hospital, 53127 Bonn, Germany; 6 Department of Cardiothoracic Surgery, University Hospital of Cologne, University of Cologne, 50923 Cologne, Germany; 7 Institute for Diagnostic and Interventional Radiology, University Hospital Dresden, 01307 Dresden, Germany; 8 Clinic for Pneumology and Allergology, Bethanien Hospital GmbH Solingen, 42699 Solingen, Germany; 9 Department of Cardiothoracic Surgery, University Hospital of Birmingham, Birmingham B15 2GW, UK; 10 Department of Radiology, University Hospital Cologne, 50937 Cologne, Germany; 11 Department of Radiology, Hospital of the Goethe University Frankfurt, 60590 Frankfurt am Main, Germany; 12 Department of Cardiothoracic Surgery, Stanford University, Palo Alto, CA 94304, USA; 13 Institute of Pathology, University of Cologne, 50923 Cologne, Germany; 14 Department of Nuclear Medicine, West German Cancer Center, University Hospital Essen, University Duisburg-Essen, 45239 Essen, Germany; 15 Department of Pneumology, West German Cancer Center, University Medical Center Essen-Ruhrlandklinik, University Duisburg-Essen, 45239 Essen, Germany; 16 Department of Medical Oncology, West German Cancer Center, University Hospital Essen, University Duisburg-Essen, 45239 Essen, Germany; 17 National Center for Tumor Diseases (NCT) West, Campus Essen, 45147 Essen, Germany

**Keywords:** pulmonary lesion, logistic scoring system, malignancy, smartphone

## Abstract

**Simple Summary:**

This study aimed to improve the precision of classifying pulmonary lesions as malignant using a new scoring system called ‘LIONS PREY’ (Lung lesION Score PREdicts malignancY). The new model, developed through the evaluation of a patient cohort from a single center, incorporates eight parameters, including age, nodule size, spiculation, solidity, size dynamics, smoking history, pack years, and cancer history. LIONS PREY demonstrates excellent precision and might facilitate decision making for multidisciplinary teams. Furthermore, it may help patients make informed decisions about surgery. The LIONS PREY app is available for free on Android and iOS devices.

**Abstract:**

Objectives: Classifying radiologic pulmonary lesions as malignant is challenging. Scoring systems like the Mayo model lack precision in predicting the probability of malignancy. We developed the logistic scoring system ‘LIONS PREY’ (Lung lesION Score PREdicts malignancY), which is superior to existing models in its precision in determining the likelihood of malignancy. Methods: We evaluated all patients that were presented to our multidisciplinary team between January 2013 and December 2020. Availability of pathological results after resection or CT-/EBUS-guided sampling was mandatory for study inclusion. Two groups were formed: Group A (malignant nodule; n = 238) and Group B (benign nodule; n = 148). Initially, 22 potential score parameters were derived from the patients’ medical histories. Results: After uni- and multivariate analysis, we identified the following eight parameters that were integrated into a scoring system: (1) age (Group A: 64.5 ± 10.2 years vs. Group B: 61.6 ± 13.8 years; multivariate *p*-value: 0.054); (2) nodule size (21.8 ± 7.5 mm vs. 18.3 ± 7.9 mm; *p* = 0.051); (3) spiculation (73.1% vs. 41.9%; *p* = 0.024); (4) solidity (84.9% vs. 62.8%; *p* = 0.004); (5) size dynamics (6.4 ± 7.7 mm/3 months vs. 0.2 ± 0.9 mm/3 months; *p* < 0.0001); (6) smoking history (92.0% vs. 43.9%; *p* < 0.0001); (7) pack years (35.1 ± 19.1 vs. 21.3 ± 18.8; *p* = 0.079); and (8) cancer history (34.9% vs. 24.3%; *p* = 0.052). Our model demonstrated superior precision to that of the Mayo score (*p* = 0.013) with an overall correct classification of 96.0%, a calibration (observed/expected-ratio) of 1.1, and a discrimination (ROC analysis) of AUC (95% CI) 0.94 (0.92–0.97). Conclusions: Focusing on essential parameters, LIONS PREY can be easily and reproducibly applied based on computed tomography (CT) scans. Multidisciplinary team members could use it to facilitate decision making. Patients may find it easier to consent to surgery knowing the likelihood of pulmonary malignancy. The LIONS PREY app is available for free on Android and iOS devices.

## 1. Introduction

Malignant neoplasms of the lung are the third leading cause of death [1]. Lung cancer is the most common cause of death due to cancer in men (24%) and the second leading cause in women (15%) [1]. In Europe, 28% of cancer deaths are attributed to lung cancer [2]. Furthermore, 70% of malignant pulmonary nodules are diagnosed in an advanced stage [3]. This leads to an unfavorable prognosis of malignant pulmonary tumors, with a relative 5-year survival rate of 21% in women and 15% in men [1]. Tobacco smoking is known to be the key risk factor for the development of lung cancer, accounting for approximately 90% of diagnosed cases [4].

The value of CT-scan screening for lung cancer has recently been evaluated in both North America (NLST trial) [5] and Europe (NELSON trial) [6]. A deep understanding and correct interpretation of the collected patient data are critical for the success of a screening protocol. Since the etiology of pulmonary lesions differ widely, and can be of malignant or benign character [7,8], every patient’s case should be critically discussed [9]. In 40–50% of all pulmonary nodules, a malignant pathology can be diagnosed either as primary lesion of the lung or as a pulmonary metastasis of extra-pulmonary cancer [10].

Scoring systems were developed to assess pulmonary nodules in a non-invasive way. They combine criteria from the patient’s medical history and radiologic parameters to determine the probability of malignancy. Today, several models have been published, including by Swensen et al. and Zhang et al. [11,12,13], but the models do not satisfy on the level of precision. A user-friendly scoring model might help to improve decision making during the management of a pulmonary nodule [14].

This work aimed to develop a new scoring system based on risk factors and radiological criteria that is simple and easy to use and yet accurately predicts the probability of malignancy. Our new model is intended to facilitate and accelerate decision making in multidisciplinary team (MDT) conferences by meeting the following four criteria: availability, simplicity, rapidity, and reliability.

## 2. Patients and Methods

### 2.1. Patient Recruitment

**Figure 1:**
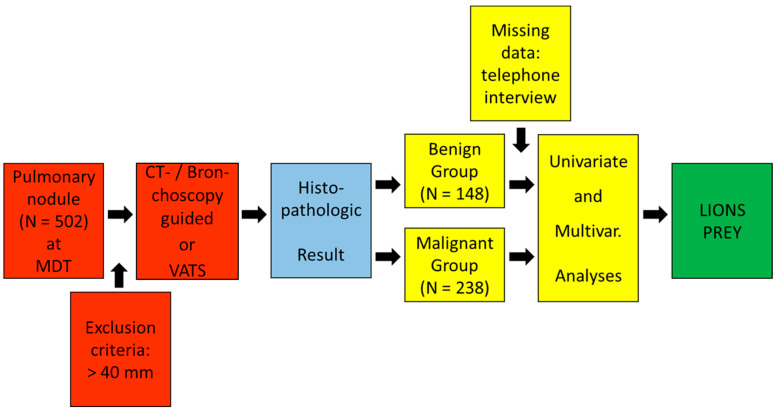
Flowchart on patient selection.

This retrospective study on prospectively collected patients’ data was registered under the number 21-1211 at the ethics committee of the University of Cologne, which waived the consent of patients on 9 April 2021. The patient recruitment was conducted at our institution between January 2013 and December 2020. We recruited all patients with a solitary pulmonary nodule that were presented to our MDT. Patients with a pulmonary mass (>40 mm) were excluded from this study. This allowed us to include solitary pulmonary nodules (SPN) beyond the normal size, as they are commonly defined as a single lung opacity of a size up to 30 mm. All patients underwent either VATS resection of the pulmonary lesion or CT-/bronchoscopy-guided tissue sampling for histological analysis. Pathological specimens and results were required for study inclusion. We formed two study groups from the patient collective. Group A were patients with histological proof of malignant pulmonary nodule. Group B were patients with histological proof of benign pulmonary nodule after resection. The recruitment process is displayed in Figure 1.

### 2.2. Data Collection

To develop this new scoring system, eleven patient-related and eleven radiological parameters were collected (Table 1). Several of them are used in different combinations in other score models to calculate the probability of malignancy in pulmonary nodules [11,12,13]. We examined five parameters with regard to malignancy that are not included in other scores. These include the GOLD stage of chronic obstructive pulmonary disease (COPD), the maximum standardized uptake value (SUVmax) of positron emission tomography (PET-CT), the size dynamics of a pulmonary nodule, and the lung function parameters FEV1 and TLCO.

Patient data were acquired from the electronic hospital information system ORBIS (Dedalus HealthCare GmbH, Bonn, Germany). Thoracic CT scans with a maximum slice thickness of 1.5 mm were evaluated in the IMPAXX EE image archiving program (Dedalus HealthCare GmbH, Bonn, Germany). Pulmonary nodules were assessed according to established radiological criteria [15] and in accordance with the Fleischner criteria [16]. Missing patient data were resolved by a telephone call with the patient or his/her relatives before the start of the analysis.

### 2.3. Score Development and Calculation

In order to identify the best parameters for LIONS PREY (Lung lesION Score PREdicts malignancY), we first performed a univariate analysis using stepwise (backward) binary logistic regression (Table 1). In a second step, all significant parameters were included in a multivariate analysis using a multinominal logistic regression to construct the scoring system (Table 2). In this way, specific beta coefficients and a constant were finally obtained. The calculation of LIONS PREY provides a predicted risk of malignancy as a percentage. The score is calculated using the following formula:

Predicted malignancy = exp (β_0_ + β_1_ × x_1_ + β_2_ × x_2_ + … + β_i_ × x_i_)/(1 + exp (β_0_ + β_1_ × x_1_ + β_2_ × x_2_ + … + β_i_ × x_i_))

Here, β_0_ is the constant of the logistic regression equation and β_i_ is the coefficient of a variable (Table 2). Among the eight LIONS PREY parameters, we have two different kinds of variables. There are four continuous variables (age, nodule size, size dynamics, and pack years) and four categorical variables (spiculation, solidity, smoking history, and cancer history). The X_i_ of the continuous variables corresponds to the given value of this variable. For the categorical variables, X_i_ = 1 when this category of the variable is present and X_i_ = 0 when it is absent. 

Considering the eight LIONS PREY parameters, X = −6.964 + (0.028 × age) + (2.163 × smoking history) + (0.017 × pack years) + (0.726 × cancer history) + (0.046 × nodule size) + (0.818 × spiculation) + (1.141 × solidity) + (0.556 × size dynamics). 

In the following equation, we show the LIONS PREY calculation for a 56-year-old smoker (42 pack years) without previous cancer, who presented to our MDT with a pulmonary nodule of 21 mm that was spiculated and non-solid but grew 4 mm within the last three months. In this case, X = −6.964 + (0.028 × 56) + (2.163 × 1) + (0.017 × 42) + (0.726 × 0) + (0.046 × 21) + (0.818 × 1) + (1.141 × 0) + (0.556 × 4). The predicted malignancy = exp (X)/(1 + exp (X)) = 81.6%. Hence, the pulmonary nodule of this patient has an approximately 82% probability of being malignant. 

### 2.4. Statistical Analysis

Discrimination and calibration, which are the standard methods for performance assessment of scoring systems, were used in this study [17]. 

For calibration analysis, we used the Grunkemeier method [18], which compares the observed malignancy with that predicted by the model within severity strata. In our study, the observed/expected ratio (O/E ratio) represents the ratio of the actual observed (O) pulmonary malignancy to the predicted (expected) malignancy (E) by the score model.

The discrimination, which is the ability of a scoring model to differentiate between a benign and a malignant pulmonary nodule, was evaluated with receiver-operating-characteristic (ROC) curves [19]. In order to compare our new scoring system with an established model, we assessed the widely used Mayo score on our study population [11]. The comparison of ROC curves was performed using the method of DeLong et al. [20].

Furthermore, we calculated the overall correct classification (OCC), which is the ratio of correctly predicted benign and malignant pulmonary nodules to the total number of analyzed cases, and assessed the mean squared error (MSE) as average squared difference between the value observed and the value predicted from the model.

Generally, continuous scale data are presented as mean ± standard deviation and analyzed by Kolmogorov–Smirnov test for normal distribution and the two-tailed Student’s *t*-test for independent samples. Categorical variables were analyzed using the Pearson’s chi-squared or Fisher exact test. All independent variables with significant differences in univariate analysis were subject to a multinominal regression analysis for further evaluation. Multinomial differences are described by odds ratio (OR) and 95% confidence interval (CI). A *p*-value < 0.05 was considered statistically significant throughout this study. All statistical analyses were performed using the program IBM SPSS Statistics version 27 (Armonk, NY, USA).

## 3. Results

### 3.1. Patient Characteristics

**Figure 2:**
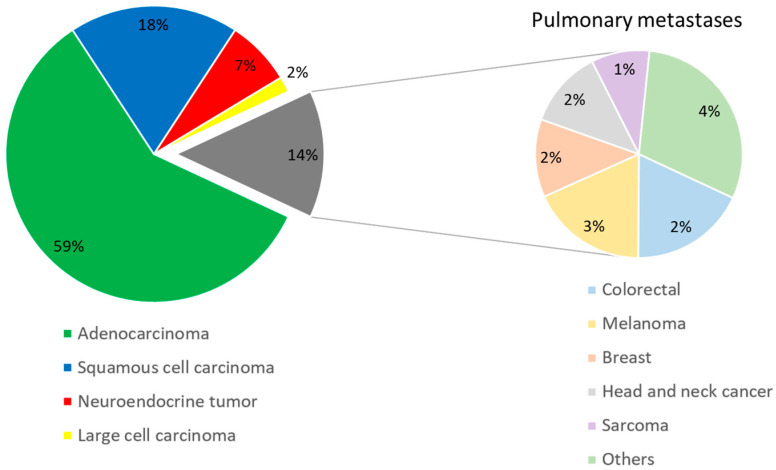
Histology of malignant tumors.

Initially, a total of 502 patients were presented to our MDT. Due to our predefined exclusion criteria, 116 patients were not considered. Finally, 386 patients were included in this study. Of the study population, 238 (62%) patients had a malignant pulmonary nodule (proven by VATS: 78%; proven by biopsy: 22%) and 148 (38%) patients had benign histology (proven by VATS: 100%). Males represented 54% of patients (*n* = 208). The gender distribution was similar in both groups (*p*-value = 0.714). The mean patient age was 63.4 ± 11.8 years. In the benign group, patients, on average, were three years younger than in the malignant group (*p*-value = 0.030). At the time of presentation to the MDT, 92% of patients with a malignant pulmonary nodule had a positive history of smoking with a combined average of 35 ± 19 pack years. In contrast, patients in Group B with benign histology smoked significantly less (smoking history: 44%, *p*-value < 0.0001; pack years: 21 ± 19, *p*-value < 0.0001). It was found that 14% of the malignant lesions were metastases from extra-pulmonary tumor manifestation. Among them, metastases of colorectal cancer, malignant melanoma, breast cancer, and head and neck cancer were most frequent (Figure 2). Positive history of cancer was reported by 35% of patients in Group A and by 24% in Group B (*p*-value = 0.025).

### 3.2. Radiologic Parameters

A total of 584 thoracic CT scans were evaluated in this work. Three-month control CT scans were available for 54% of patients (*n* = 107) in Group A and for 46% of patients (*n* = 91) in Group B. The size of pulmonary nodules was significantly (*p*-value < 0.0001) larger in Group A (21.8 ± 7.5 mm) compared to Group B (18.3 ± 7.9 mm). Furthermore, benign lesions grew significantly slower within three months (Group A: 6.4 ± 7.7; Group B: 0.2 ± 0.9; *p*-value < 0.0001). Both spiculation and solidity were more frequently observed in malignant pulmonary nodules (73% vs. 42% and 85% vs. 63%, respectively; *p*-value < 0.0001). 

Table 1 and Table 2 summarize the detailed results of patient characteristics and radiological parameters. Furthermore, the results of the univariate and multivariate analyses are presented in these tables.

### 3.3. Performance Assessment

**Figure 3:**
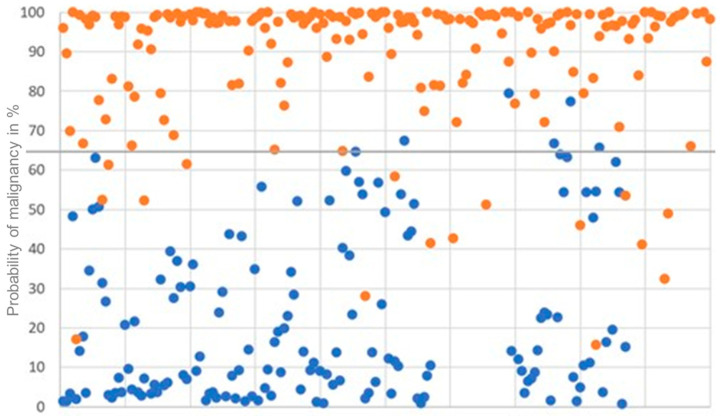
Individual score values of patients as a percentage: Group A (orange) and B (blue).

The average probability of malignancy in the study population, according to our new scoring system, was 61.5%. The mean LIONS PREY score of patients with a malignant pulmonary nodule was 85.0%. In the benign lesion group, the mean score was 23.8%. We identified a highly significant difference of the mean scores between the groups with a *p*-value of <0.0001. Based on our results, we defined the cutoff for separating the two groups as 65%. Figure 3 shows the distribution of the LIONS PREY scores in the patient population.

**Figure 4:**
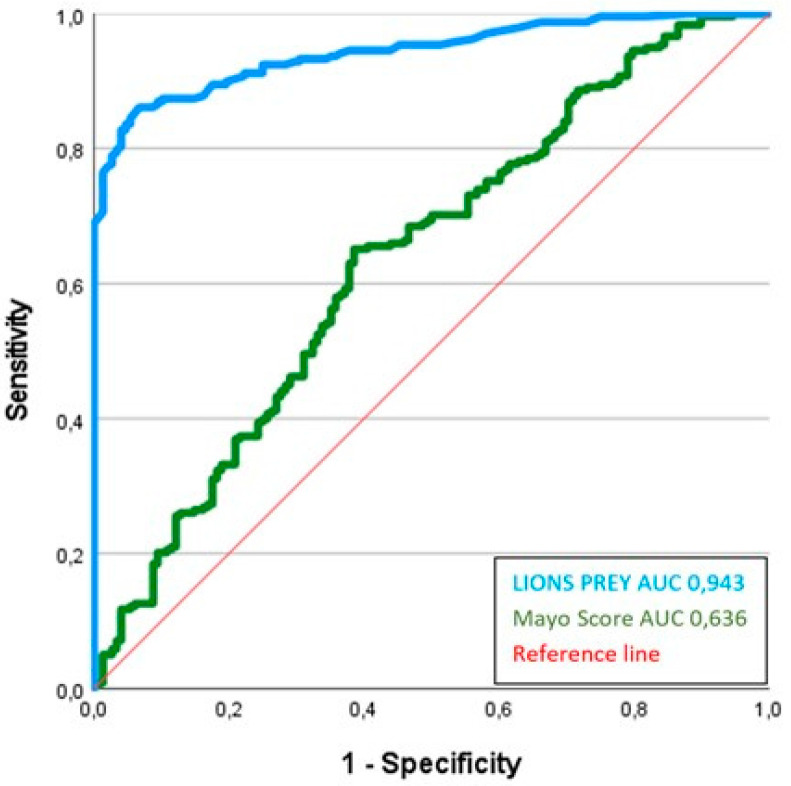
ROC curve of LIONS PREY and Mayo score.

LIONS PREY had excellent discrimination with an AUC of 0.94 (95% confidence interval: 0.92–0.97). In contrast, the Mayo score achieved an AUC of 0.64 (95% CI: 0.58–0.69); the *p*-value after DeLong analysis was 0.013. Figure 4 demonstrates the ROC curve of LIONS PREY and Mayo score. LIONS PREY reached an overall correct classification of 96.0% and a mean squared error of 0.0523.

The calibration of our new model showed an O/E-ratio of 1.1.

## 4. Discussion

### 4.1. Precision and Limits of a Scoring Model

When using scoring models, it is important to know both the precision and, particularly, the limitations of the model. It should be noted that a scoring system is a measurement method and can be subject to various disturbing effects and systematic errors. If the user is aware of these and can draw the correct conclusions from the scores’ results, misinterpretation can be widely avoided [21]. 

In a scoring model, a complex situation is reduced to a single value. Detailed information might get lost during this process. The clear advantage, however, lies in an objective and reproducible result, which makes communication between medical staff easier. It allows an objective assessment that is independent of the physician’s experience and emotions. 

The weighting of parameters is always executed in the same predefined way. Therefore, a scoring model might not fit in every individual situation. Nevertheless, the large variety of scoring models that are used daily in medical practice demonstrates their proven utility. Scores represent a supplementary tool to the clinical assessment of a patient [22].

### 4.2. The Clinical LIONS PREY Parameters

When developing a new scoring system, the selection of parameters can be consensus-based or based on statistical analysis. Both approaches focus on selecting routinely collected, clinically relevant parameters. Parameters that are incorporated into a score should be detectable independent of the investigator [22].

Patient *age* is a parameter that can be evaluated easily and has high clinical relevance. Generally, young patients have a lower risk of developing lung cancer than older patients. In patients below 35 years of age, lung diseases are more often benign [23]. With increasing age, the risk of lung cancer rises and reaches a maximum mortality rate between 80 and 84 years [2]. In our study, age is shown to be a significant parameter in relation to the probability of pulmonary malignancy.

Tobacco smoking is the most important risk factor in the development of lung cancer. Thus, the duration of consumption and the number of cigarettes smoked are proportional to the risk of cancer [24]. Nevertheless, no threshold exists below which exposure is harmless [25]. During the development of LIONS PREY, we decided to enhance the contribution of smoking by integrating both the patient’s *smoking history* (at least 100 cigarettes in their lifetime) and the number of *pack years* (number of packs of cigarettes smoked per day multiplied by the number of years the person has smoked).

During the medical history interview, patients are regularly asked about previously diagnosed cancers. In the case of a previous malignant disease, the risk of second malignancy increases by up to 20%, depending on the age and prognosis of the initial tumor [26]. If cancer therapy was already necessary in childhood, the risk of malignancy is increased 3–6-fold [26]. Therefore, we considered the patient’s *cancer history* as a parameter in LIONS PREY.

### 4.3. The Radiological LIONS PREY Parameters

By current definition, the assessment of pulmonary nodules is primarily based on the evaluation of CT scans. Here it is important to define examiner-independent criteria that do not require deep radiologic expertise.

The *size* of a pulmonary nodule can be determined quickly and easily. With increasing diameter, the likelihood of malignancy rises [26,27]. However, no differentiation of biological behavior can be made based on size alone.

Hence, the doubling rate of tumor volume is assessed. If tumor doubling occurs within 20 to 400 days, a malignant process must be assumed [23,26]. However, determining the volume of a tumor is time-consuming and needs expertise. We therefore examined the *size dynamics* by use of a simple 2D diameter measurement in contrast to a sophisticated 3D volumetric analysis of a pulmonary nodule within three months. This parameter, which is derived from clinical practice, showed high significance, with a *p*-value of <0.0001. Even without the benefit of size dynamics, LIONS PREY can also be calculated if only one CT scan is available. In this case, the score is automatically calculated in the app with only seven parameters.

*Spiculation* of the pulmonary lesion is associated with a malignant process in 97% of cases and is thus an established feature in its evaluation [28]. A smooth boundary of a nodule is more likely benign, but malignancy, particularly metastases, may be present in 20–30% of cases [28]. The fact that LIONS PREY takes metastases into account is a clear advantage over competitor products. This means that our app can depict a real-world scenario and is therefore much more suitable for everyday use as a high level of pre-selection is unnecessary.

The assessment of a pulmonary nodule concerning its *solidity* can be made with a simple look at the CT scan images. The same applies to whether or not calcification is present within a lesion. However, solidity of a pulmonary lesion is indicative of malignancy in only 7–15% of cases [29]. We consider two factors as reasons why *solidity* nevertheless emerged as a significant parameter in our analysis. First, LIONS PREY detects not only primary malignancies of the lung but also metastases of extra-pulmonary tumors, which often present as a solid on the CT scan [30,31]. Notably, we found metastasis in a relevant proportion (14%) of our patients with malignant pulmonary tumors (Figure 2). Additionally, by combining calcification and the solid character of a lesion into only one parameter, we achieved an odds ratio of 3.130 (95% CI: 1.443–6.790) and high significance in our logistic regression analyses. Consequently, we included this parameter in our new system.

### 4.4. Exclusion of Potentially Relevant Parameters

Accurate determination of Hounsfield units is difficult for untrained personnel and can lead to misinterpretation if performed inaccurately [23]. In our study, there was a wide variation in the estimation of Hounsfield units. Also, the collection of this parameter is not suitable for rapid clinical use. Since we found no significance in univariate analysis with respect to malignancy probability, the Hounsfield unit was not considered as a scoring parameter.

Although the localization of a pulmonary nodule is easy to determine in a CT scan, and the literature describes a frequent occurrence of malignant lesions in the right upper lobe, a reliable differentiation of biological behavior based on location is not possible [23]. In this study, no significance was shown with regard to localization. In contrast to the Mayo score, this parameter was not considered in LIONS PREY.

Positron emission technology (PET) is an established tool to assess pulmonary nodules. Usually, 18-fluoro-2-deoxy-d-glucose (FDG) is used and the degree of glucose turnover in the corresponding tissue is measured. This turnover is reported as the standardized uptake value (SUV). Malignant processes have been shown to have increased metabolic activity and thus demonstrate a high SUV [32]. In addition to PET, a CT scan can be performed. This allows for better localization of the region with increased metabolic turnover as well as the identification of surrounding anatomic structures [33]. FDG-PET/CT has a sensitivity of approximately 97% and a specificity of 78% for malignant pulmonary lesions with an SUVmax of >2.5 [33]. We investigated the SUVmax as a possible parameter in our new score. Since FDG-PET/CT is frequently used for the staging of lung cancer but not as a basic tool in routine diagnostics of a pulmonary nodule, SUVmax was not integrated as a parameter in LIONS PREY.

### 4.5. What Are the Strengths of LIONS PREY?

In addition to the previously described advantages of a scoring system, LIONS PREY has specific features that support its important role in everyday clinical practice. These advantages are combined in our LIONS PREY app under the themes of availability, simplicity, rapidity, and reliability. 

**Figure 5:**
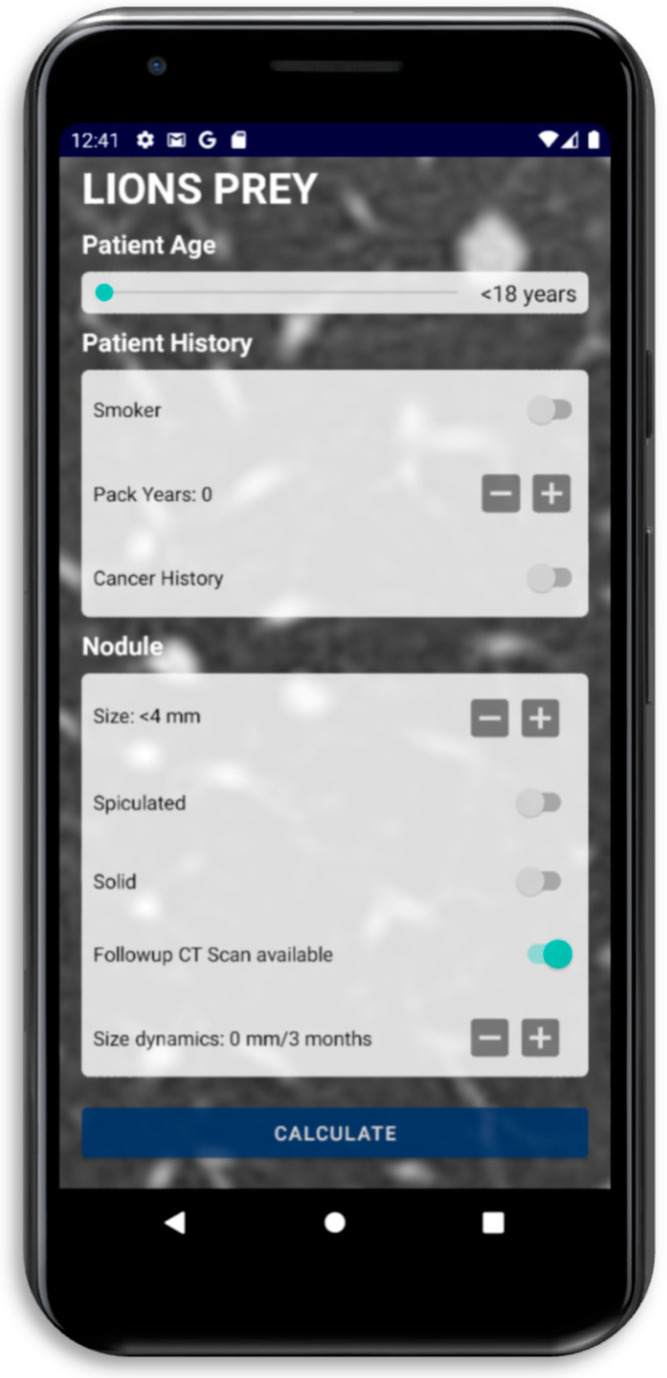
Image of LIONS PREY app.

The availability criterion includes the distribution of the scoring system free of charge as an Android and iOS app (Figure 5) and its applicability to these ubiquitous devices. Every physician can carry our scoring system as an app in her or his coat pocket. The mobile device does not require data transfer to calculate the score and can be easily used during a MDT meeting or while examining a patient. When using the app, no protected health information is collected or exposed.

Simplicity is an important characteristic of a successful scoring system. All parameters of LIONS PREY are easy to collect and are part of the routine diagnostics performed after the detection of a pulmonary nodule. They can be collected easily during the patient interview and based on CT-scan images. All eight score parameters are categorical or continuous and do not require further calculation. In particular, it was of importance to us that no parameters were included that are examiner-dependent or vary from one to the other medical institution. 

The importance of rapidity becomes apparent especially during MDT meetings. In these meetings, where different specialists come together to decide on the further treatment of a patient, a fast evaluation and assessment of the case is desirable. Often there is not enough time to read long findings. In these situations, LIONS PREY is explicitly suitable, as entering the necessary data and calculating the score take only seconds. Simple and fast applicability was a goal in creating this model. We hope that the advantage of rapidity will lead to a wide acceptance of our scoring system in clinical practice.

For the treating physicians, as well as for the patients themselves, reliability regarding the biological behavior of a pulmonary nodule is highly important since further treatment is based on it. Hence, a robust performance on the institutional level (good calibration) and on the individual patient’s risk level (good discrimination) is needed. LIONS PREY fulfills both requirements since our study design has considered all possible causes of malignant and benign pulmonary lesions. Hence, solid statements can be made when evaluating the possibility of malignancy and can be presented as a percentage.

The correct use of LIONS PREY in daily practice is highly relevant. Based on our results, a recommendation for histological confirmation can be made for pulmonary nodules that are evaluated with a probability of malignancy > 65%. As can be seen in Figure 3, a malignant histology must be expected when the probability of malignancy in LIONS PREY is >80%. In this case, a resection should be offered to the patient.

### 4.6. Validation of LIONS PREY by Comparison with the Mayo Score

In order to validate LIONS PREY internally, we also calculated the Mayo score [11], which has been established for years, and tested it on our patient population. Both models are logistic scoring systems that attempt to accurately estimate the probability of malignancy of a pulmonary nodule in a percentage. They are based on different parameters. The Mayo score, published by Swensen et al. in 1997, includes three radiological and three patient-related parameters [11]. Some of the parameters established in the Mayo score were considered in LIONS PREY. Common parameters of the two scores are age, smoking history, nodule size, cancer history, and spiculation. In addition, the Mayo score includes the parameter of localization in the upper pulmonary lobe. LIONS PREY, on the other hand, incorporates the parameters of solidity, size dynamics, and pack years.

The original publication presents the Mayo score with an AUC of 0.83. In the validation setting, the AUC was 0.80 [11]. Applying the Mayo score to our patient population resulted in an AUC of only 0.64 (95% CI: 0.58–0.69). In contrast, LIONS PREY has an AUC of 0.94 (95% CI: 0.92–0.97). The DeLong analysis shows a significant (*p*-value: 0.013) superiority of the LIONS PREY method over the Mayo score (Figure 4).

This significant difference in discrimination may be particularly associated with the double consideration of the risk factor of smoking (smoking history and pack years). Furthermore, we especially consider the size dynamics, which is a clear criterion for malignancy, as an advantage of our new model.

### 4.7. Limitations

One of the study limitations is the retrospective nature of our work which might lead to a bias that affects *p*-values. Due to our retrospective data collection, it was not possible to include the parameter of SUVmax in our new score. There were not enough data for this on the side of the benign patients’ group as they often did not receive a PET/CT scan. Nevertheless, this value is important for the assessment of the biological behavior of a pulmonary nodule. It is normally evaluated during the staging of lung cancer [34] and is part of the Herder model [35]. In contrast to the Brock algorithm, which refers to screening findings, our scoring system was developed on incidental findings within the lung parenchyma. Lastly, we have to state that development and validation of LIONS PREY was executed on the same cohort of patients. Hence, the model might be overfitted to our cohort. In the future, LIONS PREY needs to be validated prospectively on external data. 

## 5. Conclusions

LIONS PREY appears to be a reliable logistic scoring system for the prediction of malignancy in pulmonary nodules, whose parameters can be collected examiner-independently and with little effort. A smartphone application is available for calculating the malignancy probability, allowing for quick and easy calculation. Due to its good discrimination and calibration, it is a suitable tool for use in MDT meetings. In such settings, it might significantly improve decision making. Should screening recommendations for lung cancer be expanded in the future, the number of incidentally detected pulmonary nodules will increase. Consequently, there is an ever-increasing need to determine the biological behavior of these pulmonary lesions. LIONS PREY is an ideal support for these daily functions in our clinics.

## Figures and Tables

**Table 1:** Baseline characteristics and results of univariate analysis.

Predictors	All Patients (*n* = 386)	Malignant (*n* = 238)	Benign (*n* = 148)	*p* Value
Age in years (mean ± SD)	63.4 ± 11.8	64.5 ± 10.2	61.6 ± 13.8	0.021
Male (%)	53.9	54.6	52.7	0.713
FEV1 (mean ± SD)	77.1 ± 21.6	81.1 ± 21.4	71.6 ± 12.7	0.162
TLCO (mean ± SD)	78.8 ± 47.0	78.1 ± 53.7	80.7 ± 21.4	0.718
COPD (%)	61.1	63.9	56.8	0.164
History of cancer (%)	30.8	34.9	24.3	0.030
Family history of cancer (%)	15.7	11.3	20.3	0.249
Family history of lung cancer (%)	3.4	1.3	6.1	0.074
Current or former smoker (%)	73.6	92.0	43.9	<0.0001
Pack years (mean ± SD)	29.8 ± 20.1	35.1 ± 19.1	21.3 ± 18.8	<0.0001
Quitting smoking (%)	24.2	22.3	28.7	0.473
Diameter in mm (mean ± SD)	20.4 ± 7.8	21.8 ± 7.5	18.3 ± 7.9	<0.0001
Spiculation (%)	61.1	73.1	41.9	<0.0001
Clear border (%)	38.9	26.9	58.1	<0.0001
Solid (%)	76.4	84.9	62.8	<0.0001
Upper lobe (%)	60.6	61.3	59.5	0.712
Emphysema (%)	28.5	34.5	18.9	0.061
Calcification (%)	2.6	0.5	6.1	0.010
Nodule count	1.2 ± 0.7	1.2 ± 0.6	1.3 ± 0.8	0.229
Hounsfield units (mean ± SD)	−21.7 ± 117.0	−19.8 ± 131.6	−24.7 ± 117.0	0.709
Dynamics in mm/3 months (mean ± SD)	4.0 ± 6.8	6.4 ± 7.7	0.2 ± 0.9	<0.0001
SUVmax (mean ± SD)	5.7 ± 6.5	9.3 ± 6.0	1.3 ± 0.4	<0.0001

**Table 2:** Results of multivariate analysis and beta-coefficients.

Predictors	*p*-Value	Beta Coefficient	Odds Ratio	95% CI
Age in years	0.054	0.028	1.029	1.0–1.059
Diameter in mm	0.051	0.046	1.047	1.0–1.097
Spiculation	0.024	0.818	2.267	1.114–4.613
Solid	0.004	1.141	3.130	1.443–6.790
Dynamics in mm/3 months	<0.0001	0.556	1.744	1.511–2.014
Current or former smoker	<0.0001	2.163	8.695	3.497–21.622
Pack years	0.079	0.017	1.017	0.998–1.036
History of cancer	0.052	0.726	2.068	0.993–4.305
Constant		−6.964		

## Data Availability

The data presented in this study are available on request from the corresponding author.
